# Analysis of growth factors in serum and induced sputum from patients with asthma

**DOI:** 10.3892/etm.2014.1759

**Published:** 2014-06-04

**Authors:** HUI ZOU, QIU-HONG FANG, YING-MIN MA, XUE-YAN WANG

**Affiliations:** 1 Department of Allergy, Beijing Shijitan Hospital, Capital Medical University, Beijing 100038, P.R. China; 2 Department of Pulmonary and Intensive Care Unit, Beijing Shijitan Hospital, Capital Medical University, Beijing 100038, P.R. China

**Keywords:** asthma, phenotype, epidermal growth factor, basic fibroblast growth factor, platelet-derived growth factor, vascular endothelial growth factor

## Abstract

Epidermal growth factor (EGF), basic fibroblast growth factor (bFGF), the AA and BB isoforms of platelet-derived growth factor (PDGF) and vascular endothelial growth factor (VEGF) are involved in the pathogenesis of airway inflammation in asthma. In the present study, the associations between asthmatic phenotypes and the expression levels of these mediators in induced sputum and serum were investigated. A total of 62 asthmatic patients were divided into eosinophilic or neutrophilic phenotypes by cytological classification of the induced sputum. In addition, patients were classified according to lung function (FEV1/FVC >70% or FEV1/FVC <70%) and asthma severity (mild, moderate or severe). The concentrations of EGF, bFGF, PDGF-AA, PDGF-BB and VEGF in the serum and induced sputum were measured using sandwich enzyme immunoassays. VEGF levels in the serum and induced sputum were higher in patients with an eosinophilic phenotype compared with those with a neutrophilic phenotype. In addition, VEGF expression was higher in patients with an FEV1/FVC value of <70% as compared with patients with an FEV1/FVC value of >70%. Furthermore, the levels of VEGF were higher in patients with severe asthma compared with the patients with mild and moderate asthma. There were no statistically significant differences observed with regard to EGF, bFGF, PDGF-AA and PDGF-BB levels among the various phenotypes. Therefore, the observations of the present study indicated that increased VEGF expression in the serum and induced sputum of patients may be associated with eosinophilic airway inflammation, severe airflow limitation and the severity of asthma.

## Introduction

Asthma is a type of heterogeneous, chronic airway disease with a variety of phenotypes. The disease is characterized by reversible airway obstruction and airway hyperreactivity (AHR) (1). Phenotypes of asthma may be classified according to clinical features or divided into eosinophil or neutrophil types according to inflammatory cell types by immunopathogenesis (1–3). Differentiating the phenotypes of asthma clinically is important since the natural course, the prognosis of the disease and the treatment responses are not the same among the different phenotypes (1,2).

Growth factors have an important role in airway inflammation and remodeling in asthma. Epidermal growth factor (EGF) and its receptor are highly expressed in bronchial epithelial cells (4–6). Not only is EGF widely expressed in airway epithelium, gland and smooth muscle, the growth factor is also involved in the pathological and physiological processes of airway remodeling in asthma (6). EGF directly promotes the proliferation of smooth muscle in airway remodeling (7,8). In addition, the activated receptor of EGF and the receptor kinase of platelet-derived growth factor (PDGF) can enhance the proliferation of airway smooth muscle, epithelial and goblet cells (9). The levels of basic fibroblast growth factor (bFGF) in the bronchoalveolar lavage fluid (BALF) of patients with asthma have been demonstrated to be significantly higher compared with non-asthmatic patients, and were further increased following allergen exposure, indicating that bFGF is associated with airway remodeling in asthma (10). Furthermore, vascular endothelial growth factor (VEGF) levels in the serum and sputum of stable and acute exacerbation asthma patients have been shown to be increased. The expression of VEGF and its receptor are closely associated with the formation of new blood vessels, indicating that VEGF may also be involved in airway remodeling (11).

EGF, bFGF, PDGF and VEGF have important roles in airway inflammation, airway obstruction and AHR during the pathogenesis of asthma. Thus, clinical indicators that are easily obtained, practical, operable, repeatable and meaningful are required in clinical practice to aid the assessment of airway inflammation and the severity of asthma, thereby guiding the selection of targeted therapies and evaluating the prognosis for the patients. Few systematic studies investigating correlations among levels of EGF, bFGF, PDGF and VEGF in the serum and induced sputum with different clinical phenotypes of asthmatic patients have been performed. Thus, in the present study, outpatients and inpatients with asthma were recruited from the Beijing Shijitan Hospital (Beijing, China) between 2010 and 2011, and the levels of EGF, bFGF, PDGF and VEGF in the serum and induced sputum were detected. Correlations between growth factors and the phenotypes of asthmatic patients were then analyzed with the aim of producing results that may be useful for the prevention and treatment of severe asthma.

## Subjects and methods

### Case collection

A total of 62 outpatients and inpatients from the Beijing Shijitan Hospital, diagnosed with an acute attack of bronchial asthma, were recruited between 2010 and 2011. All patients complied with the diagnostic criteria in the Prevention and Treatment Guidelines of Bronchial Asthma prepared by the Asthma Study Group under the Respiratory Disease Branch of the Chinese Medical Association in 2008 (12). All patients had presented with recent gasping, breathlessness, dyspnea and coughing to varying degrees. Patients with chronic obstructive pulmonary disease, or complicated with other active, acute or chronic lung diseases, severe autoimmune diseases, therioma, coronary heart disease and hypertension were excluded from the study. Pregnant and lactating females were also excluded. Patients received standardized treatment, which included the administration of short-acting β-agonists as required, inhaled corticosteroids plus long-acting β-agonists and leukotriene modifiers. Horizontal comparisons of age, gender, disease history, lung function, total immunoglobulin E levels, eosinophil and neutrophil counts in the induced sputum, as well as EGF, bFGF, PDGF and VEGF levels in the serum and induced sputum, were conducted for all the patients. The levels of growth factors in the serum and induced sputum were detected in nine patients following one month of treatment. The study was conducted in accordance with the Declaration of Helsinki and with approval from the Ethics Committee of Beijing Shijitan Hospital. Written informed consent was obtained from all the participants.

### Sputum induction and processing

Sputum induction was performed as recommended by the Respiratory Disease Institute of the Chinese Medical Association (13). The patients inhaled 400 μg salbutamol via a metered-dose inhaler prior to induction. All participants were instructed to wash their mouth thoroughly with water. Patients then inhaled 3% saline at room temperature for 15 min, nebulized using an ultrasonic nebulizer at maximum output. Patients were then encouraged to cough deeply. The dosage was increased to 4% saline ultrasonic atomizing inhalation for 7 min if the patient did not produce enough sputum, which was further increased to 5% saline for 7 min if required. The sputum sample was diluted with phosphate-buffered saline solution containing dithiothreitol (final concentration, 1 mmol/l) and was centrifuged at 400 × g for 10 min. The cell pellet was then resuspended. Slides were prepared using a cytospin and were stained with hematoxylin and eosin for the differential eosinophil and neutrophil counts. The supernatant was stored at −70°C for subsequent analysis.

### Measurement of growth factors

Concentrations of the growth factors were determined in the serum and sputum samples at the same time. EGF, bFGF, VEGF, PDGF-AA and PDGF-BB levels were measured in duplicate using sandwich enzyme immunoassays (R&D Systems, Minneapolis, MN, USA), in accordance with the manufacturer’s instructions.

### Spirometry

All subjects underwent a spirometric assessment (Master Screen PFT; Jaeger, Wuerzburg, Germany) to measure the forced expiratory volume in 1 sec (FEV1) and the forced vital capacity (FVC). The results were compared with reference values.

### Statistical analysis

SPSS 17.0 software (SPSS, Inc., Chicago, IL, USA) was used for statistical analysis and data are expressed as the mean ± standard deviation. One-factor analysis of variance was used for the comparison of means among multiple groups, while the χ^2^ test was used for the comparison of classification data. P<0.05 was considered to indicate a statistically significant difference.

## Results

### Clinical characteristics of the patients

Clinical characteristics of the patients are presented in Table I. Patients were classified according to eosinophil and neutrophil phenotypes (cytological classification of the induced sputum), lung function (FEV1/FVC >70% and FEV1/FVC <70%) and the severity of asthma (mild, moderate and severe). No statistically significant differences were observed among the various groups in terms of gender, onset age (<40 years), smoking, allergic rhinitis disease history and positive family history of asthma. However, eosinophils in the peripheral blood collected from patients with an eosinophil phenotype were higher compared with the patients with a neutrophil phenotype (Table I).

### Growth factors in the serum and induced sputum

In 62 separate samples of serum and induced sputum, the levels of EGF, PDGF-AA and VEGF in the induced sputum were significantly higher compared with that in the serum. By contrast, the levels of bFGF and PDGF-BB in the serum were higher compared with that in the induced sputum (Table II).

### Growth factors in the serum and induced sputum of asthmatic patients with different clinical phenotypes

Concentrations of VEGF in the serum and induced sputum of patients with asthma of an eosinophil phenotype were higher compared those of a neutrophil phenotype. In addition, VEGF levels were higher in patients with an FEV1/FVC value of <70% as compared with those with an FEV1/FVC value of >70%. Furthermore, VEGF levels were higher in patients with severe asthma when compared with those with moderate and mild asthma. However, no statistically significant differences were observed between EGF, bFGF, PDGF-AA and PDGF-BB in the serum and induced sputum of patients with asthma (Tables III and IV).

### Variation of growth factors in the serum and sputum prior to and following treatment

A total of nine cases were followed-up, and the levels of growth factors in the serum and sputum were analyzed following one month of treatment; however, no statistically significant differences were observed among the levels of growth factors, despite the clinical symptoms of the patients improving (Tables V and VI).

## Discussion

Growth factors play an important role in airway inflammation and remodeling in asthma. In the present study, associations between the levels of growth factors in the serum and induced sputum with clinical phenotypes of asthma were investigated. The results from the present study demonstrated that EGF, bFGF, PDGF-AA, PDGF-BB and VEGF were all detected in the serum and induced sputum of asthmatic patients. Concentrations of VEGF in the serum and induced sputum of asthmatic patients with an eosinophil phenotype were higher compared with those with a neutrophil phenotype. In addition, patients with an FEV1/FVC value of <70% had higher levels of VEGF compared with patients with an FEV1/FVC value of >70%, and patients in the severe group had higher levels of VEGF compared with those in the moderate and mild groups. However, no statistically significant differences were observed with regard to EGF, bFGF, PDGF-AA and PDGF-BB levels in the serum and induced sputum of asthmatic patients. These observations indicate that the levels of VEGF may be associated with an eosinophil phenotype, airway flow limitation and the severity of asthma.

The main sources of VEGF are peripheral blood eosinophils (14,15), eosinophils in the BALF (16) and other cells in the airways, including epithelial cells, mast cells, myofibroblasts and smooth muscle cells (17). Several studies have demonstrated that VEGF levels in the serum and induced sputum of children with asthma are increased (18,19). In the present study, adult patients with acute asthma were found to have a higher eosinophil count in the peripheral blood with an eosinophil phenotype as compared with a neutrophil phenotype (Table I). The concentrations of VEGF in the serum and induced sputum of asthmatic patients with an eosinophil phenotype were higher compared with those with a neutrophil phenotype, indicating that the increased levels of VEGF in the serum and induced sputum is associated with an eosinophil phenotype and eosinophil airway inflammation.

The National Heart Lung and Blood Institute American Working Group (20) proposed that key research on asthma should focus on vasculature, since airway vascular remodeling and tissue edema are important factors in the underlying mechanisms of airway inflammation and the physiological abnormalities observed in asthma. VEGF plays an important role in airway inflammation, angiogenesis and vascular permeability, subepithelial collagen deposition, airway smooth muscle hyperplasia and airway physiological abnormalities (20,21). In addition, VEGF may lead to an increase in mucosal and submucosal vascular bed permeability, resulting in the thickening of the airway wall. Therefore, mucosal edema can significantly affect airway function (22,18). Previous studies have also demonstrated that patients with severe asthma exhibit a higher level of angiogenesis compared with patients with mild and moderate asthma (23,24). In accordance with these observations, the present study demonstrated that the concentrations of VEGF in the serum and induced sputum of asthmatic patients with an FEV1/FVC value of <70% were higher compared with those with an FEV1/FVC value of >70%. Furthermore, the VEGF levels were higher in the severe group compared with the moderate and mild groups, indicating that VEGF may be associated with the severity and restricted airflow of asthma. Therefore, VEGF may cause airway mucosa edema and thickening of the airway wall, resulting in the irreversible airflow limitation of airway function and the exacerbation of asthma severity via airway inflammation, angiogenesis and vasopermeability.

A previous study identified that inhaled corticosteroids reduced the expression of growth factors in asthma, with the level of VEGF in the induced sputum of asthmatic patients shown to decrease significantly after one year of corticosteroid treatment (25). In addition, after eight weeks of corticosteroid treatment, the level of VEGF in the induced sputum of patients with asthma was shown to decrease significantly (17). In children with an acute attack of asthma, the level of VEGF in the induced sputum was shown to decrease significantly following six weeks of inhaled corticosteroid treatment (19). However, in the present study, no statistically significant differences in the levels of growth factors in the serum and induced sputum were found prior to and following one month of inhaled corticosteroid treatment in the nine cases that underwent follow-up; although the levels of EGF, bFGF, PDGF-AA and VEGF presented a decreasing trend following treatment. These results may have been caused by an insufficient sample size, too short treatment time or that there was no distinction between the different phenotypes of the patients.

In conclusion, the results of the present study demonstrated that the concentrations of VEGF in the serum and induced sputum of asthmatic patients with an eosinophil phenotype were higher compared with those with a neutrophil phenotype. In addition, the concentration of VEGF was higher in patients with an FEV1/FVC value of <70% compared with those with an FEV1/FVC value of >70%. The levels of VEGF in the severe group were also higher compared with those in the moderate and mild groups. These observations indicate that VEGF levels may be associated with an eosinophil phenotype, airway flow limitation and the severity of asthma. Thus, the results of the present study are significant for the future prevention and treatment of severe asthma.

## Figures and Tables

**Table I:** Clinical characteristics of the asthmatic patients with various phenotypes.

Characteristics	Neutrophil phenotype (n=48)	Eosinophil phenotype (n=14)	FEV1/FVC >70% (n=24)	FEV1/FVC <70% (n=38)	Mild (n=38)	Moderate (n=10)	Severe (n=14)	P-value
Age, years	55.93±14.59	66.71±9.46	50.90±17.03	60.95±13.21	51.83±14.18	65.00±12.33	67.71±12.96	-
Male gender, n (%)	16 (33)	2 (14)	2 (8)	16 (42)	12 (32)	6 (60)	0	-
Disease course, years	21.91±17.77	17.00±16.84	16.30±14.55	26.34±19.55	18.33±15.57	23.38±20.81	34.29±16.94	-
Onset age <40 years, n (%)	24 (50)	10 (71)	12 (50)	22 (58)	22 (58)	4 (40)	8 (57)	-
Smoking, n (%)	16 (33)	2 (14)	2 (8)	16 (42)	12 (32)	2 (20)	4 (29)	-
Allergic rhinitis, n (%)	32 (67)	4 (29)	16 (67)	20 (53)	26 (68)	6 (60)	4 (29)	-
Family history, n (%)	22 (46)	2 (14)	8 (33)	16 (42)	16 (44)	6 (60)	2 (14)	-
Eosinophils, %	4.68±3.33	10.06±9.74^a^	6.96±5.69	4.90±4.45	5.03±4.76	8.76±7.62	5.57±4.16	<0.05^a^
tIgE, U/ml	253.31±155.66	550.38±439.37	492.99±354.77	275.04±134.55	103.44±109.25	480.98±360.94	569.04±495.07	-
FEV1, litre	56.72±18.62	76.40±32.69	83.00±12.52	50.13±16.94^b^	60.63±22.60	59.25±17.19	42.33±6.66	<0.01^b^
FEV1/FVC, %	58.14±11.25	63.20±18.42	75.50±4.04	54.18±9.82^c^	59.30±14.52	59.25±10.05	53.33±2.31	<0.01^b^

Data for age, disease course, tIgE, eosinophils, FEV1 and FEV1/FVC are expressed as the mean ± standard deviation.

a Eosinophil phenotype vs. neutrophil phenotype;

b FEV1/FVC<70% vs. FEV1/FVC>70%.

tIgE, total immunoglobulin E; FEV1, forced expiratory volume in 1 sec; FVC, forced vital capacity.

**Table II:** Range of growth factors detected in the serum and induced sputum of asthmatic patients.

Variable	Serum concentration (n=62)	Sputum concentration (n=62)	aP-value
EGF, pg/ml	7.47±6.72 (0.147263–96.23054)	118.16±100.31 (0.946999–1548.367)	<0.05
bFGF, pg/ml	85.15±35.60 (23.67197–172.0809)	0.91±0.82 (0.140295–2.899649)	<0.01
PDGF-AA, ng/ml	0.62±0.10 (0.007405–5.402607)	81.76±53.66 (0.727473–221.6614)	<0.01
PDGF-BB, pg/ml	19363.66±11541.76 (1970.83–43662.48)	64.41±60.00 (0.628064–299.136)	<0.01
VEGF, pg/ml	78.81±63.11 (0.989691–477.4135)	438.30±298.01 (45.64021–6151.269)	<0.01

Data are presented as the mean ± standard deviation.

a Serum concentration vs. sputum concentration.

EGF, epidermal growth factor; bFGF, basic fibroblast growth factor; PDGF, platelet-derived growth factor; VEGF, vascular endothelial growth factor.

**Table III:** Levels of growth factors in the serum of asthmatic patients with various phenotypes.

Phenotypes	n	EGF, pg/ml	bFGF, pg/ml	PDGF-AA, ng/ml	PDGF-BB, pg/ml	VEGF, pg/ml	P-value
Eosinophil phenotype	14	7.96±5.10	78.13±7.83	0.91±0.49	13066.17±4938.55	149.44±68.67	<0.05^a^
Neutrophil phenotype	48	7.35±3.78	86.90±7.28	0.55±0.20	20121.84±2128.29	61.15±12.59	
FEV1/FVC >70%	24	24.92±11.53	77.90±10.58	0.80±0.30	19878.75±3289.15	54.64±70.77	
FEV1/FVC <70%	38	6.90±3.77	89.75±8.63	0.63±0.29	18539.43±2767.17	134.66±138.60	<0.05^b^
Mild	38	10.58±5.46	81.92±44.79	0.67±0.24	19130.14±12371.94	73.83±73.15	
Moderate	10	10.48±6.98	85.79±18.61	1.20±0.46	23422.01±11627.44	60.94±57.82	
Severe	14	1.14±0.81	93.53±22.69	0.39±0.21	19365.14±1238.26	165.14±104.66	<0.05^c^

Data are presented as the mean ± standard deviation.

a Eosinophil phenotype vs. neutrophil phenotype;

b FEV1/FVC <70% vs. FEV1/FVC >70%;

c Severe vs. moderate and mild.

EGF, epidermal growth factor; bFGF, basic fibroblast growth factor; PDGF, platelet-derived growth factor; VEGF, vascular endothelial growth factor; FEV1, forced expiratory volume in 1 sec; FVC, forced vital capacity.

**Table IV:** Levels of growth factors in the induced sputum of asthmatic patients with various phenotypes.

Phenotype	n	EGF, pg/ml	bFGF, pg/ml	PDGF-AA, ng/ml	PDGF-BB, pg/ml	VEGF, pg/ml	P-value
Eosinophil phenotype	14	21.08±10.47	0.64±0.15	81.94±20.28	58.18±32.23	449.36±72.04	<0.05^a^
Neutrophil phenotype	48	142.43±58.47	0.99±0.17	81.71±10.33	65.97±12.74	235.54±72.04	
FEV1/FVC>70%	24	145.45±110.53	1.06±0.21	98.26±11.79	64.25±11.71	227.90±182.42	
FEV1/FVC<70%	38	125.80±53.88	0.81±0.21	80.00±14.09	76.95±19.60	449.92±366.79	<0.05^b^
Mild	38	158.91±155.01	0.87±0.78	80.14±48.99	66.32±59.84	372.93±241.81	
Moderate	10	67.61±53.05	1.03±0.86	105.15±71.03	53.45±38.72	446.95±198.46	
Severe	14	111.17±104.61	0.92±0.77	92.94±61.33	100.81±90.15	623.26±458.35	<0.05^c^

Data are presented as the mean ± standard deviation.

a Eosinophil phenotype vs. neutrophil phenotype;

b FEV1/FVC <70% vs. FEV1/FVC>70%;

c Severe vs. moderate and mild.

EGF, epidermal growth factor; bFGF, basic fibroblast growth factor; PDGF, platelet-derived growth factor; VEGF, vascular endothelial growth factor; FEV1, forced expiratory volume in 1 sec; FVC, forced vital capacity.

**Table V:** Levels of growth factors in the serum prior to and following treatment.

Growth factors	Prior to treatment (n=9)	Following treatment (n=9)	P-value
EGF, pg/ml	1.23±1.19	0.98±0.37	
bFGF, pg/ml	106.12±54.18	102.87±34.62	
PDGF-AA, ng/ml	0.33±0.30	0.11±0.10	>0.05
PDGF-BB, pg/ml	11486.84±7946.09	13896.12±5887.62	
VEGF, pg/ml	12.35±11.79	7.85±6.89	

Data are presented as the mean ± standard deviation. EGF, epidermal growth factor; bFGF, basic fibroblast growth factor; PDGF, platelet-derived growth factor; VEGF, vascular endothelial growth factor.

**Table VI:** Levels of growth factors in the induced sputum prior to and following treatment.

Growth factors	Prior to treatment (n=9)	Following treatment (n=9)	P-value
EGF, pg/ml	19.68±19.04	15.68±11.92	
bFGF, pg/ml	1.13±0.58	1.09±0.37	
PDGF-AA, ng/ml	43.56±36.20	14.52±12.16	>0.05
PDGF-BB, pg/ml	38.29±26.49	46.32±19.63	
VEGF, pg/ml	74.10±64.74	47.10±39.34	

Data are presented as the mean ± standard deviation. EGF, epidermal growth factor; bFGF, basic fibroblast growth factor; PDGF, platelet-derived growth factor; VEGF, vascular endothelial growth factor.
